# Targeting Mitochondrial Dysfunction in Neurodegenerative Diseases: Expanding the Therapeutic Approaches by Plant-Derived Natural Products

**DOI:** 10.3390/ph16020277

**Published:** 2023-02-13

**Authors:** Xiaoyue Zhang, Longqin Wang, Bowen Li, Jiayan Shi, Jia Xu, Minlan Yuan

**Affiliations:** 1 State Key Laboratory of Biotherapy and Cancer Center, West China School of Basic Medical Sciences & Forensic Medicine, West China Hospital, Sichuan University, Collaborative Innovation Center for Biotherapy, Chengdu 610041, China; 2 School of Medicine, Ningbo University, Ningbo 315211, China; 3 Mental Health Center of West China Hospital, Sichuan University, Chengdu 610041, China; 4 Huaxi Brain Research Center, West China Hospital of Sichuan University, Chengdu 610041, China

**Keywords:** plant-derived natural products, mitochondria, neurodegenerative diseases, neurotherapeutics, polyphenols, sulforaphane, resveratrol

## Abstract

Mitochondria are the primary source of energy production in neurons, supporting the high energy consumption of the nervous system. Inefficient and dysfunctional mitochondria in the central nervous system have been implicated in neurodegenerative diseases. Therefore, targeting mitochondria offers a new therapeutic opportunity for neurodegenerative diseases. Many recent studies have proposed that plant-derived natural products, as pleiotropic, safe, and readily obtainable sources of new drugs, potentially treat neurodegenerative diseases by targeting mitochondria. In this review, we summarize recent advances in targeting mitochondria in neurotherapeutics by employing plant-derived natural products. We discuss the mechanism of plant-derived natural products according to their mechanism of action on mitochondria in terms of regulating biogenesis, fusion, fission, bioenergetics, oxidative stress, calcium homeostasis, membrane potential, and mitochondrial DNA stability, as well as repairing damaged mitochondria. In addition, we discuss the potential perspectives and challenges in developing plant-derived natural products to target mitochondria, highlighting the clinical value of phytochemicals as feasible candidates for future neurotherapeutics.

## 1. Introduction

Neurodegenerative diseases, including Alzheimer’s disease (AD), Parkinson’s disease (PD), Huntington’s disease (HD), amyotrophic lateral sclerosis (ALS), different types of spinal cord cerebellar ataxia (SCA), Pick’s disease, and so on, refer to the structure or function of neurons decreasing or disappearing and deteriorating over time, causing dysfunction [1]. Neurodegenerative diseases are also increasing with the continuous extension of human life span. According to statistics, by 2050, the number of patients with neurodegenerative diseases will rise to 130 million [2]. Neurodegenerative diseases seriously affect the survival and life of patients and are a severe threat to human life, health, and quality of life. Unfortunately, there is no apparent therapeutic effect for neurodegenerative diseases, and drugs for specific molecular targets have not achieved the initially expected outcome [3]. Therefore, studying new therapeutic methods for neurodegenerative diseases has crucial implications. Since there is currently no disease-modifying treatment for these diseases, plant-derived chemicals attract a lot of attention as a potential treatment option because they often offer a better and safer alternative to synthetic medications [4]. In current studies, targeting mitochondria to treat neurodegenerative diseases is a novel and practical approach [5].

Mitochondria are located in neurons and reach synapses to provide energy. Involving the spinal cord and brain, the central nervous system (CNS) performs its primary function of transmitting, storing, and processing information, producing various mental activities, and controlling physical behavior. In the central nervous system, a large number of nerve cells come together to form an organic network. Neurons have evolved complex and highly branched morphologies and structures to maintain an information transmission network between the central nervous system and peripheral organs. Because of their unique structure, neurons have a unique mechanism of mitochondrial distribution and maintenance compared to other cells. Mitochondria in neuronal cells support the high energy consumption of the nervous system and provide sufficient Ca^2+^ buffer capacity, which is the main energy source of neurons [6]. Once damaged, damaged mitochondria need to be quickly repaired or eliminated. Dysfunction of mitochondria is a critical characteristic of neurodegeneration in the central nervous system by controlling cell death [7]. Mitochondrial inefficiency and dysfunction contribute to neurodegenerative diseases, as indicated by a great deal of evidence. For example, mutations in mitochondrial DNA and excessive ROS accumulation result in aging, an important causative factor in neurodegenerative diseases, such as PD and ALS [8]. Therefore, targeting mitochondria provides a new opportunity for treating neurodegenerative diseases.

Most natural products were first discovered as drugs through clinical trials in the human model, which is also an ideal way to screen phenotypic drugs. Natural plants are an excellent source for multitarget medicines. The history of using plants dates back to 2600–2900 BC [9], and thus far, approximately a quarter of all prescription drugs come from plants [10]. Plant-derived natural products have the advantages of pleiotropic activity, broad activity, safety, and easy availability. Recent studies have shown that they can effectively block the toxic pathways associated with neurodegenerative diseases, which may serve as a practical potential therapeutic approach by targeting mitochondria [11]. This review summarizes recent advances in targeting mitochondria in neurotherapy using plant-derived natural products. According to the mechanism of plant-derived natural products on mitochondria, the mechanism of action was discussed from the aspects of biogenesis, fusion, fission, bioenergetics, oxidative stress, calcium homeostasis, membrane potential, mitochondrial DNA stability, and repair of damaged mitochondria. In addition, we describe future perspectives and challenges in developing plant-derived natural products to target mitochondria, highlighting the clinical value of plant-derived natural products as ideal candidates for future neurotherapeutics.

## 2. Plant-Derived Natural Products Stimulating Mitochondrial Biogenesis

Mitochondrial biosynthesis refers to the cellular plasticity adaptation process of mitochondrial proliferation, systematic synthesis, and individual synthesis during the life cycle of a cell. It is accomplished through a series of biochemical processes, such as crosstalk between mitochondria and the nucleus, coordinated expression of two genomes, protein synthesis and transmembrane transport, and subunit assembly of mitochondrial protein complexes. Mitochondria in human cells are derived from the original fertilized egg, and new mitochondria can only grow from old mitochondria and can never be generated from scratch. The average lifespan of mitochondria is only 28 days, so mitochondria inevitably change from old to new. In particular, terminally differentiated cells that no longer have the ability to divide and regenerate, such as brain neuronal cells and cardiomyocytes, rely entirely on the replacement of mitochondria to maintain a normal structure and function. Apoptosis is also inevitable; mitochondrial homeostasis is supported by two contrary signs of progress: the biogenesis of mitochondria and the elimination of impaired mitochondria by mitophagy. When apoptosis reaches a certain level, neurodegenerative diseases, such as Alzheimer’s or heart failure, occur [12,13].

**Figure 1:**
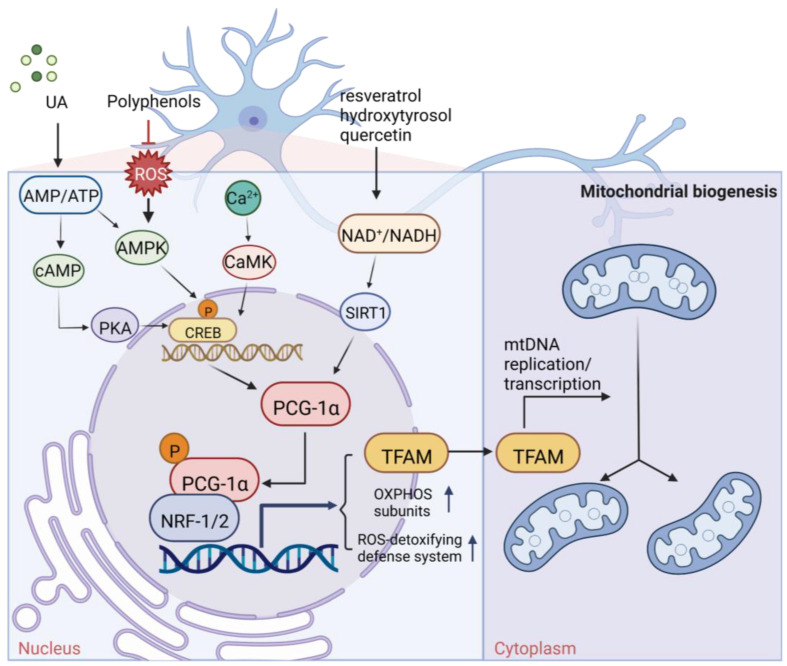
The mechanism of neuronal mitochondria biogenesis. The mitochondrial activity of neuron cells mainly depends on the expression of peroxisome proliferator activated receptor γ-coactivator 1α (PGC-1α), which is determined by AMP/ATP ratio and Ca^2+^ concentration. The NAD+/NADH ratio also participates in this pathway through Sirt1 deacetylation. Elevated concentrations of AMP and Ca^2+^ promote the expression of certain kinases and activate PGC-1α through direct and indirect phosphorylation. Subsequently, PGC-1α binds to regulatory factors nuclear respiratory factor (NRF1/2) to promote the expression of certain system proteins, such as mitochondrial transcription factor A (TFAM), which promotes the transcription and translation of mtDNA and ultimately leads to increased mitochondrial biogenesis growth. Some natural products of plant origin, such as UA, polyphenols, resveratrol, hydroxytyrosol, and quercetin, can act on the cascade to improve mitochondrial biogenesis.

The regulatory mechanism of biogenesis in mitochondria is activated step by step, and the central link of this regulatory mechanism is peroxisome proliferator activated receptor γ-coactivator 1α (PGC-1α or receptor γ) and its downstream regulatory factors nuclear respiratory factor (NRF1/2), TFAM, etc. (Figure 1). PGC-1α is responsible for the interaction between mitochondria and the nucleus because mitochondrial DNA (mtDNA) and nuclear DNA (nDNA) have two genomes that must work together to keep life alive. In the regulatory system of stepwise activation, PGC-1α activates the promoter of NRF1 to produce the NRF1 protein factor, which is very sensitive to the intracellular redox state [14,15]. NRF1 continues to activate the promoter of mitochondrial transcription factor (TFAM), produce protein factor TFAM, transport into mitochondria, bind to mitochondrial DNA (mtDNA), activate RNA polymerase, and start to perform transcription of the mitochondrial genome [16,17]. In addition, many factors, such as second messengers (cAMP, calcium, eNOS) and kinase pathways, coordinate membrane recruitment, protein import, and OXPHOS complex assembly at different levels by regulating gene expression and protein modification [18]. These courses are interrelated and work together to maintain a healthy, orderly pool of neurons, thereby preventing neurodegenerative diseases. In neuronal activity, the part of mitochondrial biogenesis cannot be ignored. When the signaling pathway in the regulation mechanism of mitochondrial germinal is disturbed, mitochondrial dysfunction may be caused, and then neurodegenerative diseases may occur. For example, the expression of PGC-1α, NRF1, and TFAM was enhanced in human pituitary oncocytoma cells and acellular adenoma cells [19].

Most of the natural products mentioned in this review that can affect gene expression in mitochondria or induce mitogenesis are of plant origin. In contrast, few acquired synthetic drugs have been affirmed as inducers of mitogenesis. For example, ginger extract (GE) and its major components, 6-gingerol and 6-chrysophanol, have been reported to facilitate mitochondrial biogenesis in mouse cells by promoting OXPHOS subunit-related proteins and activating the AMPK-PGC1α signaling pathway [20]. Ursolic acid (UA), a triterpenoid derived from various natural herbs, fruits, and vegetables, promotes mitochondrial biogenesis and ATP production, while a small amount of ROS is produced in cells. The activation of the AMPK-PGC1 pathway by ROS in mitochondria further enhances the expression of cytochrome C oxidase (COX) and uncoupling protein 3 to induce mitochondrial biogenesis [21,22]. Polyphenols, recognized antioxidants that eliminate ROS, are ubiquitous in plants. In addition to the widely believed antioxidant, anti-inflammatory, cardiovascular prevention, and other effects of polyphenols, some polyphenols are now considered to be able to regulate the regulation of mitochondrial biogenesis molecules [23]. Since mitochondrial dysfunction is directly or indirectly involved in the origin of numerous neurodegenerative diseases, including Parkinson’s disease and Alzheimer’s disease, it is highly valuable to study the restoration of mitochondrial function [24]. Polyphenols, including resveratrol in grapevine [25], hydroxytyrosol [26] in olive, and quercetin [27,28] in various plants, can induce mitochondrial biogenesis by inducing the SIRT1/PGC-1α pathway. As the main compound in flavonoids, apigenin, and similar compounds have been shown to promote adult neurogenesis by promoting neuronal differentiation or are beneficial in treating neurodegenerative diseases.

Although neurodegenerative diseases lack a clear etiology, they are caused by the interaction of various complex pathogenic factors, including mitochondrial dysfunction, oxidative stress, and neuroinflammation. Plant-derived natural products can improve many of these factors at the cellular level, making them widely used and potentially valuable for research on the treatment of neurodegenerative diseases and other related diseases in the clinic.

## 3. Plant-Derived Natural Products Regulating Mitochondrial Fusion and Fission

Mitochondrial fusion and fission is a dynamic process that remodels mitochondrial inner membrane (IMM) cristae through their interaction and plays an important role in mitochondrial transport, oxidative phosphorylation production of ATP, regulation of mitochondrial morphological number and distribution, maintenance of efficient mitochondrial function, and joint maintenance of mitochondrial homeostasis [29,30].

The mitochondrial quality control network diagram showed that multiple mitochondria fuse after a period of functioning due to a decline in their function to different degrees. The fusion process, which can share internal components, such as mtDNA and electron transport chains, is a “conservation” strategy. After a while, the relatively large mitochondria fail again, and the mitochondria begin to divide. It is important to note that this cleavage is not necessarily interrupted in the middle but can also form fragments of different lengths to eliminate discarded mitochondria. Meanwhile, this process can also recycle mitochondria by repairing mtDNA, the electron transport chain, and other parts to further realize mitochondrial biogenesis. The discarded mitochondria with decreased or even absent membrane potential can be recycled through lysosomal autophagy, such as decomposition into amino acids [31,32].

**Figure 2:**
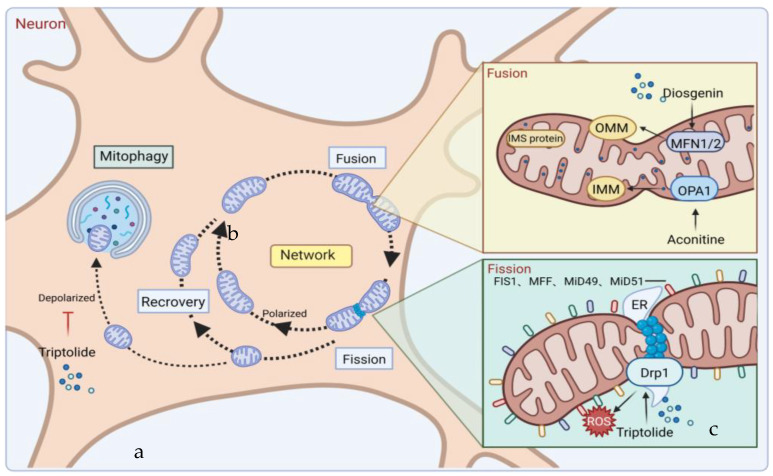
Mechanisms of mitochondrial fusion and fission and some drug effects. (**a**) During the life cycle of normal mitochondria, mitochondria are generally alone, but fusion and fission also occur. When mitochondrial fission occurs, in addition to the restoration of polarization into complete mitochondria and returning to the mitochondrial cycle, depolarization and mitochondrial autophagy also occur. (**b**) During mitochondrial fusion, optic atrophy protein 1 (OPA1) mediates the fusion of the IMM. Mitofusins 1/2 (MFN1/MFN2) mediates the fusion of OMM. OPA1 also mediates the maintenance of mitochondrial crest structure to promote OMM fusion and IMM fusion through the interaction between MFN1/2. (**c**) During mitochondrial fission, GTPase dynamic-related protein 1 (Drp1) mediates mitochondrial fission, splitting OMM and IMM, and producing two daughter mitochondria. How Drp1 interacts with ER and adaptor proteins FIS1, MFF, MiD49, and MiD51 remains to be investigated. During these processes, plant-derived natural products Aconitine and Diosgenin act on OPA1 and MFN1/2 to promote mitochondrial fusion, respectively. Triptolide acts on Drp1 to produce ROS, resulting in mitochondrial fission and mitophagy.

Inner membrane (IMM) and outer membrane (OMM) GTPases are required for the mutual fusion of the outer membrane and the inner membrane in mitochondrial fusion (Figure 2). Among these, the fusion protein mediating mammalian mitochondrial fusion is a member of the dynamin family, and optic atrophy protein 1 (OPA1) mediates mitochondrial inner membrane fusion. Mitofusins 1 and 2 (MFN1 and MFN2; OMM) mediate the fusion of the mitochondrial outer membrane [33]. OPA1 helps maintain the mitochondrial crest structure and may help anchor nucleoids to the IMM. Its deficiency or mutation may impair mitochondrial fusion, reducing mitochondrial ATP production and respiratory capacity [34]. GTPase dynamic-related protein 1 (Drp1) has the characteristic domain structure of the dynamin family, which is composed of the GTPase domain, intermediate domain (MD), and GTPase effector domain (GED) [35]. GTPase dynamic-related protein 1 (Drp1) circulates between the cytoplasm and the outer membrane of mitochondria to mediate mitochondrial fission [36]. Mitochondrial fission is a highly regulated process. Once the process of mitochondrial fission is damaged, the metabolic state of mitochondria will be changed, and then cell metabolism, proliferation, and apoptosis will be affected. Fission does not require membrane potentials but can be triggered by low membrane potentials. Mitochondrial outer and inner membrane fusion is almost coordinated, but when the membrane potential changes, internal membrane fusion is blocked, while outer membrane fusion can still occur [37]. Mitochondrial fission in neurons promotes cytoplasmic and organelle trafficking and the redistribution of cellular axons. The dysregulation of mitochondrial fusion and fission is closely related to neurodegeneration. Interestingly, the interaction of fission and fusion is used against different stress levels. In general, at low emergency levels, such as starvation, an increase in fusion or a decrease in fission is beneficial against changing stress to guard cells against the degradation response caused by excessive mitophagy [38]. However, at high emergency levels, such as apoptosis or loss of mitochondrial membrane potential, mitochondrial fission will increase, and fusion will decrease. At this point, OMA1-mediated OPA1 blocks mitochondrial fusion in human cells [39]. For example, one reason for Parkinson’s disease may be the mutations in Pink1/Parkin, which mediate mitofusin degradation, leading to the accumulation of damaged mitochondria and eventually excessive accumulation of ROS, resulting in cell killing [40].

Disorders of mitochondrial fusion and fission are closely related to neurodegenerative diseases. Previous studies have noted that the occurrence of dominant optic atrophy (DOA) and Charcot-Marie-Tooth disease (CMT) is associated with an imbalance in mitochondrial fusion and fission [41]. DOA refers to retinal ganglion cell (RGC) primary degeneration with ascending optic atrophy due to mitochondrial dysfunction and cell apoptosis caused by OPA1 and other gene mutations, resulting in vision loss [42,43]. In addition to surgery, drug therapy is mainly used for DOA, such as glucocorticoids, B vitamins, and neurotrophic factors. Charcot-Marie-Tooth (CMT), a disease characterized by progressive myasthenia and atrophy in the distal extremities, is one of the most general heterogeneous neurodegenerative diseases. According to electrophysiological characteristics, CMT can be divided into demyelinating CMT1 and axonal CMT2. Currently, no specific drugs can effectively treat CMT, mainly to support symptomatic treatment [44,45]. The occurrence of CMT and DOA is primarily due to mutations in MFN2 [46], OPA1 [47], or DRP1 [48], which leads to the blocking of mitochondrial fusion and abnormal mitochondrial transport and mitochondrial membrane potential. In addition, a recently reported mutation of the SLC25A46 gene also caused mitochondrial fusion and fission changes, leading to the occurrence of CMT and DOA [49].

The results show that triptolide is derived from the active component of *Tripterygium wilfordii*, which is a natural product of plant origin with hepatotoxicity. Triptolide may cause increased ROS generation, decreased mitochondrial depolarization, decreased mitochondria, and decreased energy generation by acting on DRP1, which may lead to mitochondrial fission and anti-mitophagy (Figure 2). The specific pathway may be a new mechanism [50]. Sulforaphane (SFN), which is widely present in cruciferous plants, can change the kinetics of mitochondrial fusion and fission by inhibiting HDAC and DNA methyltransferase, thus preventing genetic and epigenetic mutations [51]. In addition, diosgenin, which is widely distributed in various plants, has a protective effect on kidney injury by increasing the expression of mitochondrial fusion and fission-related proteins (including DRP1 and MFN2) in potatoes to reduce the imbalance of renal mitochondrial fusion and fission [52]. Other studies have shown that aconitine [53], the main toxic component of aconitine and other plants, and calenduloside E from *Pterygoides auriculata* can target OPA1 to cause mitochondrial function remodeling [54]. Because of the critical role of mitochondria in neurons, these drugs may also serve as a potential treatment modality for neurodegenerative diseases due to disturbances in mitochondrial dynamics.

In future studies, these plant-derived natural product drugs have significant potential to treat neurodegeneration caused by altered mitochondrial dynamics, including DOA and CMT.

## 4. Plant-Derived Natural Products Improve Mitochondrial Bioenergetics

The primary energy source of neuronal activity in the brain depends on glucose metabolism to generate ATP, while the primary energy consumption is neuronal signaling activities, such as action potentials. Nonsignaling neuronal activities, including protein synthesis and axonal transport, have low energy requirements [55].

Mitochondria contribute mainly to cell life activities through oxidative phosphorylation (OxPhos) [56]. Mitochondrial bioenergetics involves mitochondrial morphology, localization, kinetics, mitochondrial function, and mitochondrial homeostasis regulation [36] and then affects the function of the nervous system rich in mitochondria [57].

An apparent positive correlation was confirmed between the degree of human aging and the probability of neurodegenerative diseases. As a consequence, studies on the mitochondrial dysfunction associated with aging are of extraordinary importance. ATP produced by mitochondria is the main energy source, and processes such as electron transport chain inactivation, mitochondrial oxidative stress increase, or mitochondrial permeability change may cause mitochondrial damage, thereby affecting mitochondrial function [58]. In addition, the OxPhos system is coregulated by mitochondrial DNA (mtDNA) and nuclear DNA (nDNA) [59,60], and the coordination of these two genes plays an indispensable role in maintaining mitochondrial protein synthesis and stability. From a genetic perspective, mitochondrial diseases may result when mtDNA and nDNA mutations occur [61]. In this process, cardiolipin in the inner mitochondrial membrane takes part in maintaining mitochondrial bioenergy [62], and melatonin protects mitochondrial biofunction by preventing the oxidation of cardiolipin, thereby preventing aging-related neurodegenerative diseases [63].

**Figure 3:**
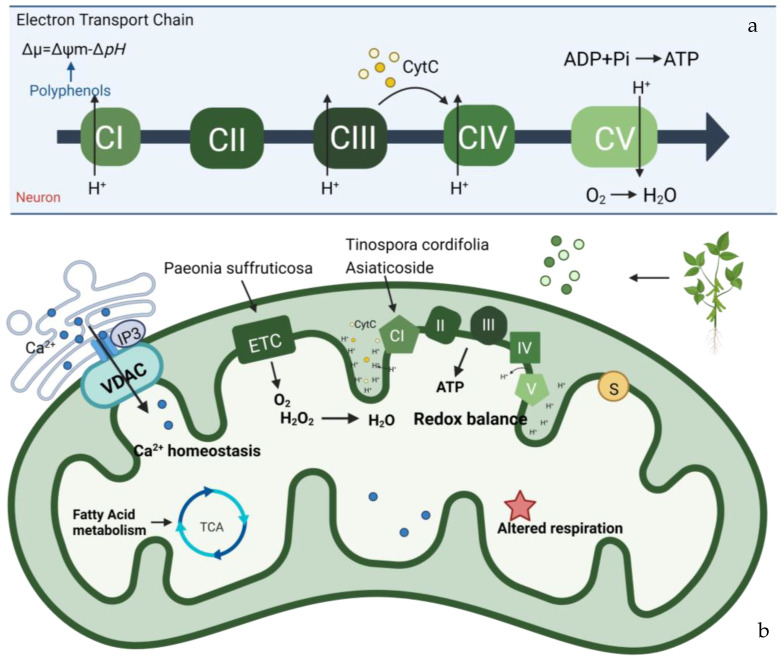
Mitochondrial bioenergy systems and plant-derived natural products improve mitochondrial productivity. (**a**) Electron transport chain (ETC). There are five polymerase complexes in the oxidative phosphorylation system: CI, CII, CIII, CIV, CV. In ETC, electrons are transferred from succinic acid to O_2_ via cytochrome C (cyt C) to produce H_2_O and ATP. The mitochondrial membrane potential (∆Ψm) balance maintains mitochondrial homeostasis and its ability to produce ATP. (**b**) Biological function of mitochondrial proteins and factors influencing the function of the energy-producing OxPhos system. Affecting the stability of mitochondrial Ca^2+^ channel, ETC channel, and other factors will have an impact on mitochondria. Some natural products from plants, such as *Paeoina suffruticosa* and *Tinospora cordifolia*, can regulate mitochondrial homeostasis.

Understanding the structure and composition of mitomicomes is crucial for the study of neuropathic diseases caused by mitochondrial damage and altered energy metabolism. OxPhos is an enzymatic reaction that produces ATP, which is essential for cellular life. In the mitochondrial respiratory chain (Figure 3), electrons removed from metabolites are gradually transferred from the low redox site to the high redox site through two electron transport chains: ubiquitin (Coenzyme Q, CoQ) and cytochrome C (cyt C). There are five types of polymerase complexes in the oxidative phosphorylation system: Complex I (CI, NADH dehydrogenase), Complex II (CII, succinate-Coenzyme Q reductase), Complex III (CIII, cytochrome C reductase (COX)), Complex IV (CIV, cytochrome C oxidase), and Complex V (CV, ATP synthase) [64,65].The main respiratory chain is the respiratory chain initiated by NADH and catalyzed by complexes I, III, and IV to generate proton gradients to transfer electrons. In contrast, the secondary respiratory chain is initiated by FADH2 and catalyzed by complexes II, III, and IV to transfer electrons from succinate to O2. The stable structure and normal function of the oxidative phosphorylation system depend on the synergistic interactions among these different complexes [66,67]. The mutated electron transport chain in the OxPhos system, which is found in some neurodegenerative diseases, may lead to its development. For instance, Leber hereditary optic neuropathy (LHON) is a disease that mainly affects the retina and the macular tract fibers of the optic papilla and leads to optic neurodegeneration. It is caused by mutations in various mtDNA-encoded subunits, which affect the stability and activity of CI and reduce ATP synthesis driven by CI substrates [68]. Mutations in any subunit of CIV have been proven to cause mitochondrial diseases [69]. For example, mutations in CIV can cause a COX10 mutation that catalyzes a block in the heme synthesis step, leading to LS and other early-onset neurological syndromes [70,71].

Parkinson’s disease (PD) is a neurodegenerative disease distinguished by a significantly decreased striatal dopamine (DA) content [72]. Clinical manifestations of PD include quiescent tremor, bradykinesia, myotonia, and postural gait disorders. The exact etiology of this pathological change is still unknown. In addition to genetic factors, environmental factors, inflammatory effects, and iron accumulation, the hypothesis of defective mitochondrial complexes associated with the electron transport chain has also received much attention [73]. Studies have shown that in PD, due to functional damage to mitochondrial complex I, a large number of reactive oxygen species (ROS) are produced during respiration. Meanwhile, CI can produce toxic hydroxyl radicals and nitric oxide and react to generate peroxynitrite, which can respond with nucleic acids to destroy nucleic acids, proteins, and lipids. Damage to the electron transport chain [74]. Natural products of plant origin play an important role in PD treatment. *Tinospora cordifolia* extract has been shown to improve mitochondrial oxidative respiration and enhance mitochondrial function by increasing the activity of mitochondrial complex I, thus treating PD [75]. *Paeonia suffruticosa* extract improves mitochondrial function by reversing the downregulation of Akt and the mitochondrial OXPHOS subunit [76]. In addition, one study showed that the administration of Asiaticoside extract, a natural glycoside derived from the Asiatica plant, effectively reduced neuronal death in a rat model of rotenone-induced Parkinson’s disease. Mechanistically, the protective effect of asiaticoside on mitochondria is achieved by protecting mitochondrial CI activity, the rate-limiting step of OxPhos [77]. The use of asiaticoside for treating neurodegenerative diseases, especially AD, has been extensively studied in clinical practice.

## 5. Plant-Derived Natural Products Preventing Mitochondrial Oxidative Stress

**Figure 4:**
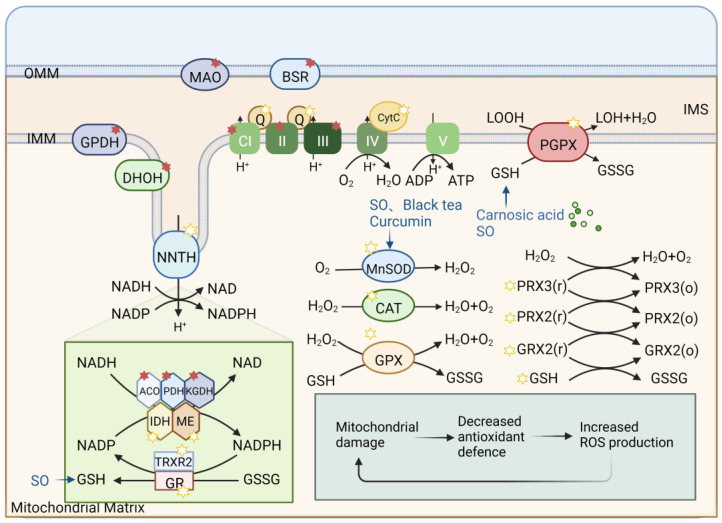
Oxidative metabolism and antioxidant system in mitochondria. The figure shows the contents and processes in mitochondria that are mainly involved in the production of ROS (red stars) and antioxidant defense systems (yellow stars). Enzymes that produce ROS are: tricarboxylic acid (TCA) cycle enzymes aconitase (ACO), α-ketoglutarate dehydrogenase (KGDH), pyruvate dehydrogenase (PDH), glycerol-3-phosphate dehydrogenase (GPDH), CI, CII, and CIII in ETC, antioxidant defense systems are: Q, CytC, d glutathione (GSH) in ETC, interacting, glutaredoxin (GRX2), thioredoxin (TRX2), peroxiredoxins (PRX3/5), etc., Enzyme manganese superoxide dismutase (MnSOD), catalase (Cat), glutathione peroxidase (GPX), isocitrate dehydrogenase (IDH) and malic enzyme (ME), etc., Oxidant membrane protein nicotinamide nucleotide transhydrogenase (NNTH), phospholipid hydroperoxide glutathione perpx (PGPX), etc. Some natural drugs have been shown to have an effect on the REDOX system.

Mitochondria act in the metabolism of ROS (Figure 4). The oxidative respiratory system of mitochondria contains many oxidoreductases, which can transfer single electrons to oxygen by potential energy to produce ROS superoxide. With the exception of the electron transport chain, ROS can also be produced through glycolysis and other pathways. Mitochondria also have an extensive antioxidant system to protect cells against oxidative stress caused by excessive ROS (Figure 4), including manganese superoxide dismutase (MnSOD), glutathione (GRX2), peroxiredoxin (PRX3/5), and nonenzymatic components, such as cyt C, glutathione (GSH), and CQ. Mitochondrial energy status partly determines the capacity and status of this antioxidant system. When the structure or function of mitochondria is damaged and the antioxidant system defense ability is reduced, the production of ROS will disrupt the redox balance [78].Mitochondrial oxidative stress can lead to the occurrence of aging, which plays a very important role in neurodegenerative diseases [8]. Therefore, enhancing mitochondrial antioxidant capacity to maintain mitochondrial homeostasis is significant for treating aging and neurodegenerative diseases.

The pathogenesis of major neurodegenerative diseases is closely related to the formation of ROS and mitochondrial dysfunction caused by oxidative stress. In this disease, changes in the antioxidant defense system (particularly the subtraction of MnSOD and GSH) and oxidative damage to proteins and DNA are common. As a specific neurodegenerative disease, AD patients always present with cognitive degeneration. Their clinical manifestations are always characterized by atrophy of the middle temporal gyrus, decreased concentration of β-amyloid 1-42 (Aβ1-42), and increased concentration of phosphorylated Tau. Oxidative damage caused by mitochondrial dysfunction is closely related to the pathogenesis of AD, which has been revealed many times. Moreover, oxidative damage often occurs in the early stage of AD and precedes the deposition of Aβ [79]. Several experimental studies have shown that some measures, such as oxidative damage treatment [80] and induction of antioxidant enzyme MnSOD failure [81], can change the concentration level and accumulation of Aβ in mouse neurons. At present, studies [82,83] have shown that it may cause AD by changing the synthesis and processing of amyloid precursor protein (APP) or tau protein by oxidative stress, and that the produced Aβ can interact with mitochondria and lead to mitochondrial dysfunction. For example, Aβ inhibits complex IV and α-ketoglutarate dehydrogenase (KGD) on the inner mitochondrial membrane (IMM) and binds to amyloid-binding alcohol dehydrogenase (ABAD) in the mitochondrial matrix. This important energy regulator maintains the estradiol/estrogen balance in neurons [84], directly or indirectly affecting mitochondrial redox status and activating signaling cascades leading to neuronal death. For example, Aβ inhibits complex IV and KGD on the inner mitochondrial membrane (IMM) and binds to amyloid-binding alcohol dehydrogenase (ABAD) in the mitochondrial matrix, directly or indirectly affecting mitochondrial redox status and activating signaling cascades leading to the death of neurons. Research has proven that APP may target the outer mitochondrial membrane (OMM) and interfere with protein input. In addition, γ-secretase complexes in the mitochondrial matrix catalyze the breakdown of APP to form Aβ and presenilin-1, which can increase the proteolytic activity of mitochondrial serine protease (HTRA2) for IAPs [85].

For the common neurodegenerative disease PD, the activity of PD complex I decreased due to overexpression of α-synuclein and inhibition of rotenone inhibitory complex I, the activity of mitochondrial kinase PINK1 with protective cell function decreased, and Parkin mutation [86] and other factors damaged mitochondrial function and promoted the occurrence of PD. Oxidative stress, for example, oxidative modification of the S-nitrogenation of parkin, will affect ubiquitination and inhibit its protective effect [87]. The mutation of J-1, a REDOX sensor [88], will also affect the regulatory effects of PTEN and PINK1, thus increasing the mutation of Parkin’s disease. In contrast, overexpression of the antioxidant system can effectively inhibit the occurrence of PD. For example, in a Drosophila Parkin mutant, the overexpression of glutathione S-transferase can effectively inhibit the damage caused by oxidative stress and then inhibit PD [89].

In recent years, several natural plant-derived ingredients have been shown to be effective in delaying or treating neurodegenerative diseases by inhibiting mitochondrial oxidative stress. For example, some medicinal plants and active ingredients can increase the levels of glutathione, superoxide dismutase, and catalase in the brain, thus exerting neuroprotective effects. Saffron, a perennial seedless herb, belongs to the Iris family of saffron. Its antioxidant properties, components, and essential associations with the treatment of neurodegenerative diseases may play an important role in the treatment of Alzheimer’s disease and Parkinson’s disease [90].

Studies have shown that asiaticoside extract not only protects CI in the OxPhos system and thus protects the mitochondrial function mentioned above but also has a strong antioxidant capacity [91]. This is done by inducing NrF2-related factors to activate antioxidant response elements (AREs) to maintain the mitochondrial redox balance and mitochondrial activity [92]. Its antioxidant properties have been clinically applied to treat neuroprotection and improve memory function in animals [93]. The activation of the Nrf2/AREs pathway provides a promising clinical therapeutic idea for enhancing mitochondrial function in neurodegenerative diseases [94,95].

In the PD model, natural plant-derived components protect against 6-hydroxydopamine-induced neurotoxicity. For example, carnosic acid induces the expression of C-glutamate-cysteine ligase catalytic subunits, superoxide dismutase, and glutathione reductase in PD models by reducing GSH [96]. Sesame oil (SO) can protect neurons by increasing the content of glutathione reductase (GR), glutathione-s-transferase (GST), GPx, catalase (CAT), GSH, and thiobarbiturate reactive substances (TBARS) and restoring the expression of MnSOD [97]. Moreover, curcumin promotes neuronal function by reducing MDA and promoting increased binding of glutathione, GPx, glutathione reductase, SOD, catalase, tyrosine hydroxylase, and D2 receptors in neurons [98].The levels of dopamine, GSH, and SOD in neurons affected by desferrioxamine are reduced, so it is applied in the treatment of PD [99]. The seed extract of *Aspergillus niger*, *Hibiscus asper* leaves, *Ginkgo biloba* extract, *Delphinium denudatum* [100], *Bacopa monniera* Linn [101], and black tea extract [102] can increase GSH content and GPX, SOD, and CAT activity.

In addition, certain common natural plant drugs may be effective in treating neurodegenerative diseases. For example, as a traditional African medical herb, the extract of Schumach leaves is commonly used as an anti-inflammatory, analgesic, and antibacterial agent and has been shown to have antioxidant potential, combating and enhancing cognitive abnormalities in Parkinson’s disease [103]. Similarly, *Withania somnifera*, a medicinal plant commonly used for anti-inflammatory and antibacterial activities in mainland India, has been shown to have anti-stress effects by reducing ROS in neurons and has the latent capacity to be a substitutive drug for the treatment of neurodegenerative diseases [104].

A better understanding of the mechanisms that regulate mitochondrial oxidative stress in neurodegenerative diseases will provide critical insights into targeting these therapeutic processes.

## 6. Plant-Derived Natural Products Modulate Mitochondrial Calcium (Ca^2+^) Homeostasis

The presence of a rapid and specific Ca^2+^ channel in the IMM, named the mitochondrial Ca^2+^ uniporter, is the reason why mitochondria play a critical role in Ca^2+^ homeostasis [105]. Excessive Ca^2+^ efflux is a severe disaster for cells and even induces apoptosis [106]. According to recent reports, plant-derived natural products may reduce the risk of neurodegenerative diseases by protecting neurons from stress-induced damage, inhibiting neuroinflammation, and altering synaptic plasticity [107].

For example, flavonoids extracted from citrus fruits and various Chinese herbal medicines have essential effects on regulating mitochondrial large-conductance calcium-regulated potassium channels [108]. It has been reported that dietary flavonoids lead to the inhibition of apoptosis and promote the survival and differentiation of neurons. These functions depend on regulating the Ca^2+^ signal in the process of mitophagy activated by gene expression [109]. Flavonoids were found to have unique antioxidant activity at lower concentrations in the brain [110,111], and some reported that their function of protecting and enhancing neurons was achieved by attenuating the inhibition of thapsigargin on sarco/ER Ca^2+^-ATPase (SERCA) pumps [112]. SERCA-mediated Ca^2+^ uptake into stores [113], which act to maintain intracellular free Ca^2+^ levels within a physiological limit.

In addition to flavonoids and their analogs, abundant photogenic natural compounds, such as quinic acid and resveratrol, can activate mitochondrial ATP synthase-dependent respiration by increasing mitochondrial Ca^2+^. Therefore, the efficiency of neuronal oxidative metabolism could be improved, and thus, we can reduce the risk of neurodegenerative diseases [112,114].

Mitochondria play a major role in apoptosis. Some studies have accelerated the efflux of Ca^2+^ from the cell membrane by knocking down the key apoptosis-inhibiting gene Bcl-2 and found that the rate of Ca^2+^ efflux was positively correlated with the proportion of apoptosis, proving that Bcl-2 gene-downregulated pore formation is a critical step in the induction of apoptosis [115]. Moreover, some natural plant-derived polyphenols, such as astaxanthin, phenolic acids, coumarin, and sesame lignans, have been shown to significantly promote the anti-apoptotic effect of neuronal cells [116,117,118,119]. These anti-apoptotic functions have been generally achieved through upregulation of Bcl-2 and Bcl-cL, downregulation of Bax and Bak, and Ca^2+^ homeostasis [106,120,121,122,123].

In addition to some herbal medicines practiced by traditional Chinese medicine for many years in treating neurodegenerative diseases, some of the same natural plant extracts primarily used in the past have recently received increasing attention. The medicinal plant *Tripterygium wilfordii* is controversial. In recent years, some researchers have believed that it has unexpectedly sound curative effects in treating various diseases, especially neurodegenerative diseases [124,125,126]. However, other reports believe that its efficacy is not worth mentioning compared to its toxicity [127]. Celastrol is an essential medical component of Tripterygium wilfordii that has a neuroprotective effect and can potentially treat neurodegenerative diseases. Celastrol inhibits abnormally activated astrocytes and prevents neuronal apoptosis and neuroinflammation [128]. Celastrol can inhibit abnormally activated astrocytes and prevent neuronal apoptosis and neuroinflammation by affecting the permeability of the mitochondrial membrane to Ca^2+^, so it has therapeutic functions in different neurodegenerative diseases, such as AD, HD, PD, and stroke [127,129].

Meanwhile, we cannot just look at how to prevent the efflux of Ca^2+^ from the mitochondria. Some excitatory neurotoxins increase the permeability of mitochondrial membranes and uptake far more than safe amounts of Ca^2+^, disrupting neuronal activity. Biapigenin, found in cereals, activates related transcription factors and enhanced Ca^2+^ efflux from mitochondria, which could reduce the Ca^2+^ burden of mitochondria and protect cells against excitotoxicity [106].

These plant-derived natural products play an essential role in modulating mitochondrial calcium (Ca^2+^) homeostasis and are promising for use in preventing and treating neurodegenerative diseases in the near future.

## 7. Plant-Derived Natural Products Sustaining Mitochondrial Membrane Potential (∆Ψm)

One of the essential causes of neurodegenerative diseases is the loss of mitochondrial function in nerve cells to varying degrees. Before signs of neuronal cell death are clear, significant changes in mitochondrial membrane integrity lead to dissipation of the transmembrane potential, and an imbalance in mitochondrial membrane potential (Δψm) will significantly affect the production capacity of mitochondrial adenosine triphosphate (ATP) [130,131], concomitantly affecting the selective permeability of mitochondrial membranes.

There are many ways to artificially interfere with the mitochondrial membrane potential, among which there are many reports on effectively using natural compounds to modulate the mitochondrial membrane potential, thereby inhibiting apoptosis. Quercetin, a plant-derived flavonoid abundant in various plants, exhibits prominent antioxidative and anti-inflammatory activities [132,133].

Polyphenols, such as morin and mangiferin (4-Glucosyl-1,3,6,7-tetrahydroxyxanthone), protect the mitochondrial membrane potential Δψm and prevent the activation of caspases in neurons, thereby inhibiting apoptosis [106].

In the previous sections, we mentioned that natural flavonoids control the ability of nerve cell mitochondria to control Ca^2+^. Nevertheless, the effects of such natural compounds on nerve cell mitochondria are far greater than that. Some researchers have used chronic ethanol induction (for 15 weeks) to build a mouse model of mitochondrial damage in central neurons and have administered a natural flavonoid called quercetin. The results showed that quercetin significantly attenuated the loss of mitochondrial membrane potential in neurons [133].

We should realize that the integrity of the mitochondrial membrane is a necessary prerequisite for the physiological function of mitochondria. Damage to the integrity of the membrane inevitably leads to a series of catastrophic consequences. Therefore, it is somewhat biased to consider the effect of natural compounds on membrane potential alone, and it should be studied together with other functions of the mitochondrial membrane.

## 8. Plant-Derived Natural Products Maintain Mitochondrial DNA Stability

**Figure 5:**
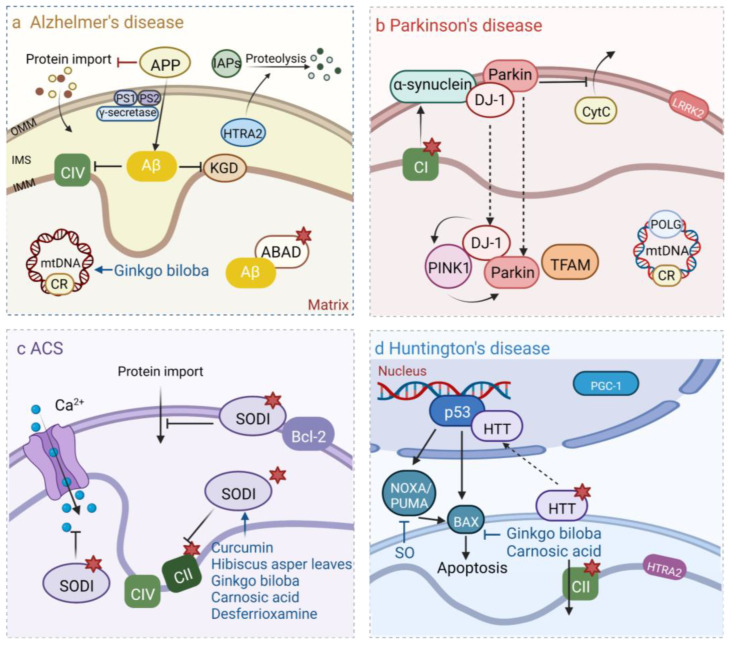
Mechanisms of mitochondria in four common neurodegenerative diseases. (**a**) In AD, Amyloid precursor protein (APP) can affect protein input by targeting OMM. APP is cleaved by γ-secretase complex to form Aβ. Aβ inhibits mitochondrial electron transport chain complex CIV and α-ketoglutarate dehydrogenase (KGD) and binds to Aβ-binding alcohol dehydrogenase (ABAD). Aβ increased the proteolytic activity of HTRA2 to IAPs. ROS were produced by both KGD and ABAD (red stars). MtDNA mutations increased in AD. (**b**) Mutations in complex I and POLG encoded by mtDNA are also pathogenic factors in PD. Overexpression of α-synuclein can impair mitochondrial function. Parkin is associated with mitochondrial transcription factor A (TFAM), which binds to OMM and inhibits the release of CytC. During oxidation, DJ-1 moves to mitochondrial IMS and stroma, and mitochondrial kinase PINK1 acts with DJ-1 and Parkin to protect cells. Kinase LRRK2 can be localized to OMM. (**c**) In ALS, the overexpression of mutant SOD1 can affect the concentration of Ca^2+^ in mitochondria, protein transport, and ATP production. Mutant SOD1 promotes the abnormal production of mitochondrial ROS and reduces the mutational activity of the neuronal mitochondrial complex CII. (**d**) In HD, the mutant HTT translocated to the neuron nucleus and promoted the transcriptional activity of p53, which activated the proapoptotic factor BAX either directly or by promoting the expression of NOXA and PUMA. In addition, HTRA2 mutations are also a cause of HD. In this process, natural plant-derived products have different effects.

In the genome, mitochondria have their own set of DNA, in addition to the nuclear genome. Human mitochondrial DNA (mtDNA) is a double-chained loop-like molecule composed of 16,569 base pairs encoding 13 peptides located in the mitochondrial inner membrane, as well as multiple RNAs and tRNAs, which are used for the transcription and translation of proteins in mitochondria and prepare for the production of ATP by oxidative phosphorylation in mitochondria [8]. In the current study, genetic mutations in mitochondrial DNA are known to give rise to multifarious diseases, most of which affect energy-intensive tissues, such as the brain and muscles [8]. Therefore, we believe that changes in mitochondrial DNA are closely related to the occurrence of neurodegenerative diseases and become more significant with age [134]. In addition, in the mtDNA mutant mouse model [135], it was concluded that mitochondrial mtDNA mutation and mitochondrial oxidative stress both affect mitochondrial function. Nevertheless, ROS did not mediate the mtDNA mutation process, and these two processes were independent but could influence each other. With the process of aging, oxidative damage to mitochondrial DNA is significantly upregulated, and its sensitivity to oxidative stress is also increased considerably. Nevertheless, at the same time, a lack of repair leads to further mitochondrial function damage. In mitochondria, mutations in mtDNA encode enzymes in antioxidant systems that also play an important role in neurodegeneration (Figure 5).

The release of some mitochondrial proteins is relevant to the apoptosis and senescence of neuronal cells. In addition, mitochondrial DNA-encoded proteins are crucial for neurodegenerative diseases. AD has been shown to be hereditary in 5–10% of cases and occurs in an early-onset, autosomal dominant manner. Amyloid precursor protein (APP) causes Aβ, which is a major component of age plaques, whereas the PS1 and PS2 A components of gamma-secretase cleavage APP produce Aβ. Aβ can bind to Aβ-binding alcohol dehydrogenase (ABAD) [136]. Inhibit the interaction of Aβ and the ABAD complex with other inducer proteins, and inhibit the proapoptotic effect of Aβ on neurons [137]. In addition, Aβ also inhibits α-ketoglutarate dehydrogenase activity [138] and interacts with the serine protease HTRA2 to degrade denatured proteins [139,140]. In PD, the gene-encoded Parkin protein acts as the ubiquitin E3 ligand, and DJ-1 and PINK can protect cells from oxidant-induced cell death. mtDNA mutations and multiple nuclear genes have been identified as risk factors for Parkinson’s disease. The Parkin protein encoded by the gene acts as ubiquitin E3 ligand, α-synuclein, and DJ-1 can induce PINK, LRRK2, HTRA2, etc. Furthermore, mutations in the complex I subunit, 12S rRNA [141], and POLG encoded by mtDNA also contribute to Parkinson’s disease. In ALS, overexpression of SOD1 after mutation can impair electron transport chain activity and Ca^2+^ concentration in mitochondria. Mutant SOD1 binds to and isolates the anti-apoptotic protein Bcl-2 [142] and can promote the abnormal oxidative stress level of mitochondria. Targeting mutant SOD1 to mitochondria can lead to the release and apoptosis of Cyt and inhibit the transport of OMM proteins [143]. The clinical manifestations of Huntington’s disease are dance-like movements and progressive cognitive dysfunction, which are inherited autosomal dominant. In HD, mutations in the mitochondrial coding sequence have been found to produce extended polyglutamine repeats, which are closely related to the disease [144].

Some natural plant-derived components play a role in neurodegenerative diseases caused by such mechanisms. Studies have shown that ginsenoside Rg1, a tetracyclic triterpenoid derivative derived from ginseng [145], acts on cells to restore mitochondrial activity in neurons by restoring upregulated Bax expression and downregulated Bcl-2 mRNA and protein expression and is clinically used in the treatment of PD [146]. In addition to increasing dopamine content in the MPTP-induced PD model, extracts of the natural plant *Paeonia suffruticosa* can also reverse the downregulation of Akt and mitochondrial OXPHOS subunits, thus protecting neurons [147]. Ginkgo biloba extract can increase the amount of dopaminergic D2 receptors in the striatum by encoding mitochondrial DNA, thus promoting neuronal activity. Black tea extract can promote mtDNA transcription translation, TH protein levels, and TH mRNA expression [102]. In addition, most of the plant components mentioned above that can affect the protein expression level in the REDOX system are influenced by mtDNA, and the two parts are inseparable from each other. Treatments based on mitochondrial DNA are being used in neurodegenerative diseases.

## 9. Perspectives and Conclusions

Neurodegenerative diseases seriously affect the survival and life of patients, and the study of the treatment of neurodegenerative diseases has extremely far-reaching and vital significance. Recently, the therapeutic effects and potential of plant-derived natural products have received attention. As the main energy source of neurons, mitochondria play a critical role in neurodegenerative diseases. Therefore, research on the mechanism of mitochondrial action in the pathogenesis of neurodegenerative diseases is helpful for the clinical understanding and application of plant-derived natural products in treating neurodegenerative diseases.

Extracts from different natural plants showed various therapeutic activities toward neurodegenerative diseases (Table 1), including beneficial effects directed toward mitochondria. In this paper, we first classified neurodegenerative diseases caused by mitochondrial functional damage caused by different mechanisms: biogenesis, fusion, fission, bioenergetics, oxidative stress, calcium homeostasis, membrane potential, mitochondrial DNA stability, etc. Then, according to the different mechanisms of disease occurrence, the related natural products from the plants were summarized. These natural products can effectively inhibit the toxic pathways related to neurodegenerative diseases as drugs. Plant-derived natural products may be an exciting new possibility in targeted mitochondrial therapy for neurodegenerative diseases and may become a potential therapeutic route in the clinic. Perhaps the field holds great promise for treating these diseases. However, the mechanisms of action of many plant extracts and their active components in neurodegenerative disease models need to be further investigated in future experiments.

## Figures and Tables

**Table 1:** Summary of plant active ingredients effective on neurodegenerative disease.

Name	Plant Origin	Function	Chemical Structures	References
Carnosic acid	*Rosmarinus officinalis* L.	1. Improve mitochondrial activity 2. Reduced lipid peroxidation 3. GSH reduction 4. Increased protein expression of Gclc, SOD, and GR 5. Reduction of the Bcl-2/Bax ratio 6. Induction of caspase 3 cleavage 7. Induction of poly (ADP ribose) PARP cleavage	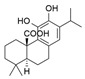	[96]
Curcumin	*Curcumalonga* L.	1. Decreased MDA 2. Increased GSH, GPx, GR, SOD, catalase TH and D2 receptor binding in brain tissue	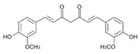	[98]
Ginsenoside Rg1	Ginseng	1. Improve mitochondrial activity 2. Promote mitochondrial biogenesis 3. Expression of Bax and Bcl-2 mRNA and protein	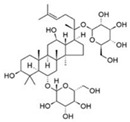	[146]
Quercetin	Peas, potatoes, broad bean leaves, apple peels, etc	1. Mitochondrial biogenesis is induced by SIRT1/PGC-1α pathway 2. Decreased protein carbonyl content	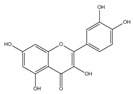	[27,28]
Desferrioxamine	Some grasses, fruit trees, etc	1. Decreased protein carbonyl content 2. Increased dopamine, GSH, and SOD levels	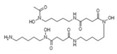	[99]
Sulforaphane	Cruciferous plants, such as broccoli, Chinese kale, carrot, etc.	1. Altering mitochondrial fusion and fission by inhibiting HDAC and DMT 2. Blocking DNA fragmentation and caspase-3 activation 3. Increased GSH levels	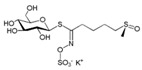	[51]
6-gingerol/6-chrysophanol	ginger	Promotion of OXPHOS subunit related proteins and activation of alpha signaling pathways promote mitochondrial biogenesis	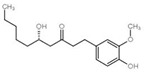	[20]
Ursolic acid (UA)	Herbs, fruits, and vegetables	1. Promote mitochondrial ATP production 2. The production of a small amount of ROS activates the AMPK-PGC1 pathway and further increases the expression of COX and uncoupled protein 3, thus inducing mitochondrial biogenesis	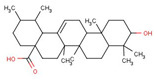	[21]
Triptolide	*Tripterygium wilfordii*	1. Acting on DRP1 leads to increased ROS generation, decreased mitochondrial depolarization, decreased mitochondrial number, and decreased ATP generation 2. Mitochondrial fission and mitochondrial autophagy 3. The specific pathway may be a new mechanism	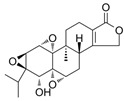	[50]
Diosgenin	Widely used in a variety of plants.	Increased expression of mitochondrial fusion and fission-related proteins (including DRP1 and MFN2)	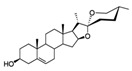	[52]
Aconitine	Sichuan black, grass black, aconite, etc	Effect of OPA1 on mitochondrial function remodeling	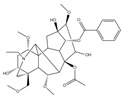	[53]
Flavonoids	A plant widely occurring in nature	1. Regulate mitochondrial large conductance calmodulating potassium channel and activate the regulation of Ca^2+^ signal 2. Attenuating the inhibition of thapsigargin on sarco/ER Ca^2+^- ATPase (SERCA) pumps 3. Inhibit neuronal apoptosis and promote neuronal survival and differentiation 4. Antioxidant activity	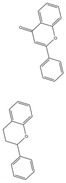	[108,109,110,112]
Resveratrol	Grapes	1. Induce mitochondrial biogenesis by inducing the SIRT1/PGC-1α pathway 2. Activate the mitochondrial ATP synthase-dependent respiration by increasing mitochondrial Ca^2+^	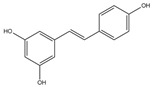	[25]
Astaxanthin	Rhodiaceae, *Chlorella*, etc.	1. Bcl2 and Bcl-cl were upregulated, while Bax and Bak were downregulated 2. Maintain Ca^2+^ homeostasis 3. Promote the anti-apoptosis of neuron cells	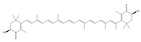	[106,116,120,121]
Biapigenin	Cereals	1. Activated related transcription factors enhanced Ca^2+^ efflux from mitochondria, which could reduce the Ca^2+^ burden of mitochondria 2. Protected cells against excitotoxicity	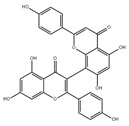	[106]
Mangiferin	Polyphenols	1. Protected the MM potential Δψm 2. Prevent the activation of caspases in neurons 3. Inhibiting apoptosis	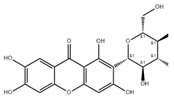	[106]
Saffron	Iris family of saffron	1. Improve mitochondrial behavioral performances 2. Suppression of a-synuclein overexpression or aggregation 3. Promote the expression of the antioxidant system	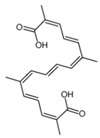	[90]
Asiaticoside	Asiaticoside	1. Mitochondria are protected by protecting the rate-limiting step of OXPHOS, CI activity 2. Effectively reduces neuronal death 3. The Nrf2/AREs pathway is activated to maintain mitochondrial Redox balance	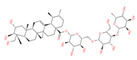	[77,92,93]
Alkaloids	Tinospora cordifolia	1. Increased mitochondrial complex I activity 2. Decreased MDA levels 3. Improve mitochondrial activity	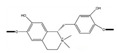	[148]
Sesame seed oil (SO)	Sesame seed	1. Increased GR, GST, GPx, CAT, GSH, and TBARS 2. Inhibit the activation of Nox2 and Cox2 3. Restored MnSOD expression	N.A.	[97]
*Paeonia suffruticosa*	Peony	1. Increased total striatal dopamine 2. Reversed downregulation of Akt and the mitochondrial OXPHOS subunits	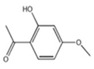	[147]
*Hyoscyamus niger* seeds	*Hyoscyamus niger* seeds	1. Attenuated motor disabilities 2. Increased level of GSH content and GPX, SOD, and CAT activities 3. Inhibiting MAO and scavenging hydroxyl free radicals	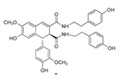	[149,150]
*Hibiscus asper* leaves	*Hibiscus asper* leaves	1. Increased SOD, GPX, and CAT activities, total GSH content 2. Reduced MDA level	N.A.	[151]
Bilobalid	*Ginkgo biloba*	1. Increased GSH content 2. Decreased generation of TBARS 3. Increased SOD and CAT activities 4. Coding mitochondrial DNA increases the number of dopaminergic D2 receptors in the striatum.	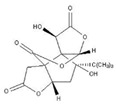	[152,153]
Norditerpenoid alkaloids	*Delphinium denudatum*	1. Decreased MDA levels 2. Increased GSH content 3. Increased SOD and CAT activities	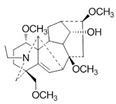	[100]
*Bacopa monnieri* (Linn.)	*Bacopa monnieri* (L.) Wettst.	1. Decreased MDA levels 2. Increased GSH content 3. Increased SOD and CAT activities	N.A.	[154]
Catechins	Black tea	1. Recovery DA-D2 receptor binding 2. Decreased MDA levels 3. Increased GSH content 4. Increased SOD and CAT activities 5. Increased TH protein level and TH mRNA expression	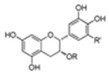	[102,155]
Hydroxytyrosol	Olive	Induce mitochondrial biogenesis by inducing SIRT1/PGC-1α pathway	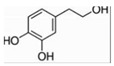	[26,156]
Schumach	Leaf of Schumach	Promote the expression of antioxidant system	N.A.	[103]
Bacoside A3	*Withania somnifera*	Reduces ROS in neurons, reduces the stress effect	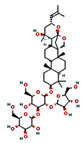	[104,157]

## Data Availability

Not applicable.
